# Supplementary figures and images for: SIV and Mycobacterium tuberculosis synergy within the granuloma accelerates the reactivation pattern of latent tuberculosis
Source: PLoS Pathog. 2020 Jul 30;16(7):e1008413. doi: 10.1371/journal.ppat.1008413 (PMC7419014; doi:10.1371/journal.ppat.1008413)

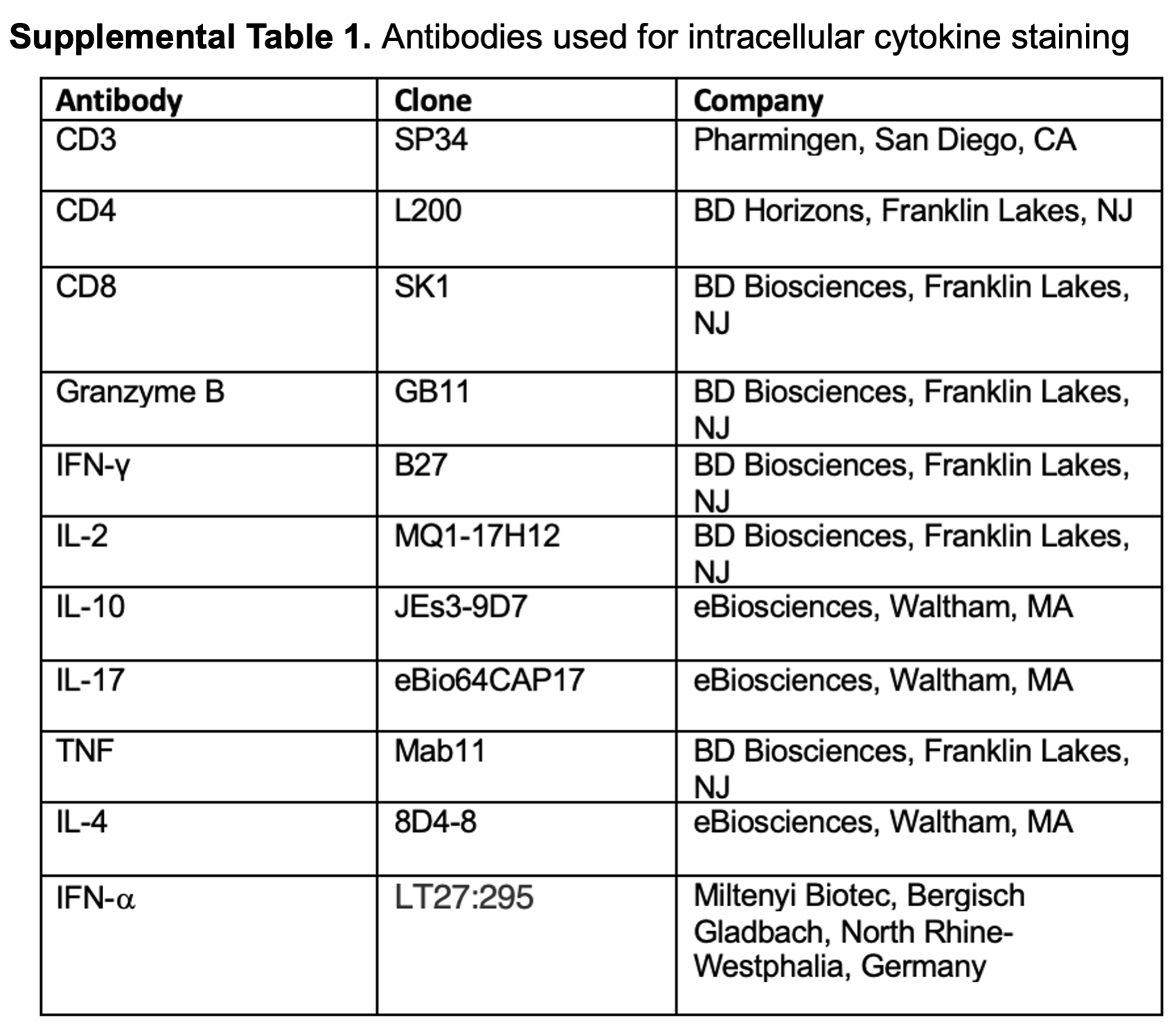

Supplement: S1 Table — (TIFF) [file ppat.1008413.s001.tiff]

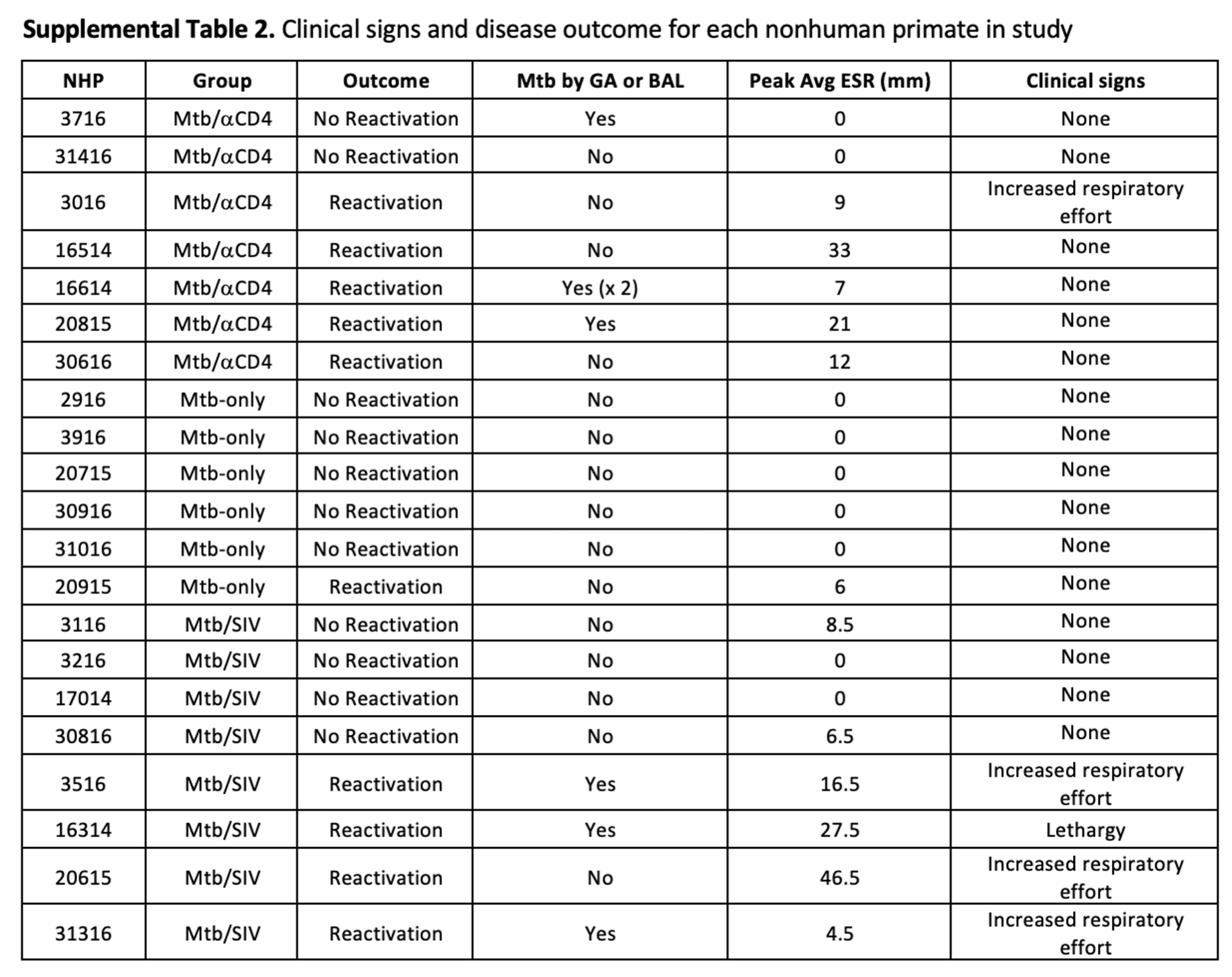

Supplement: S2 Table — (TIFF) [file ppat.1008413.s002.tiff]

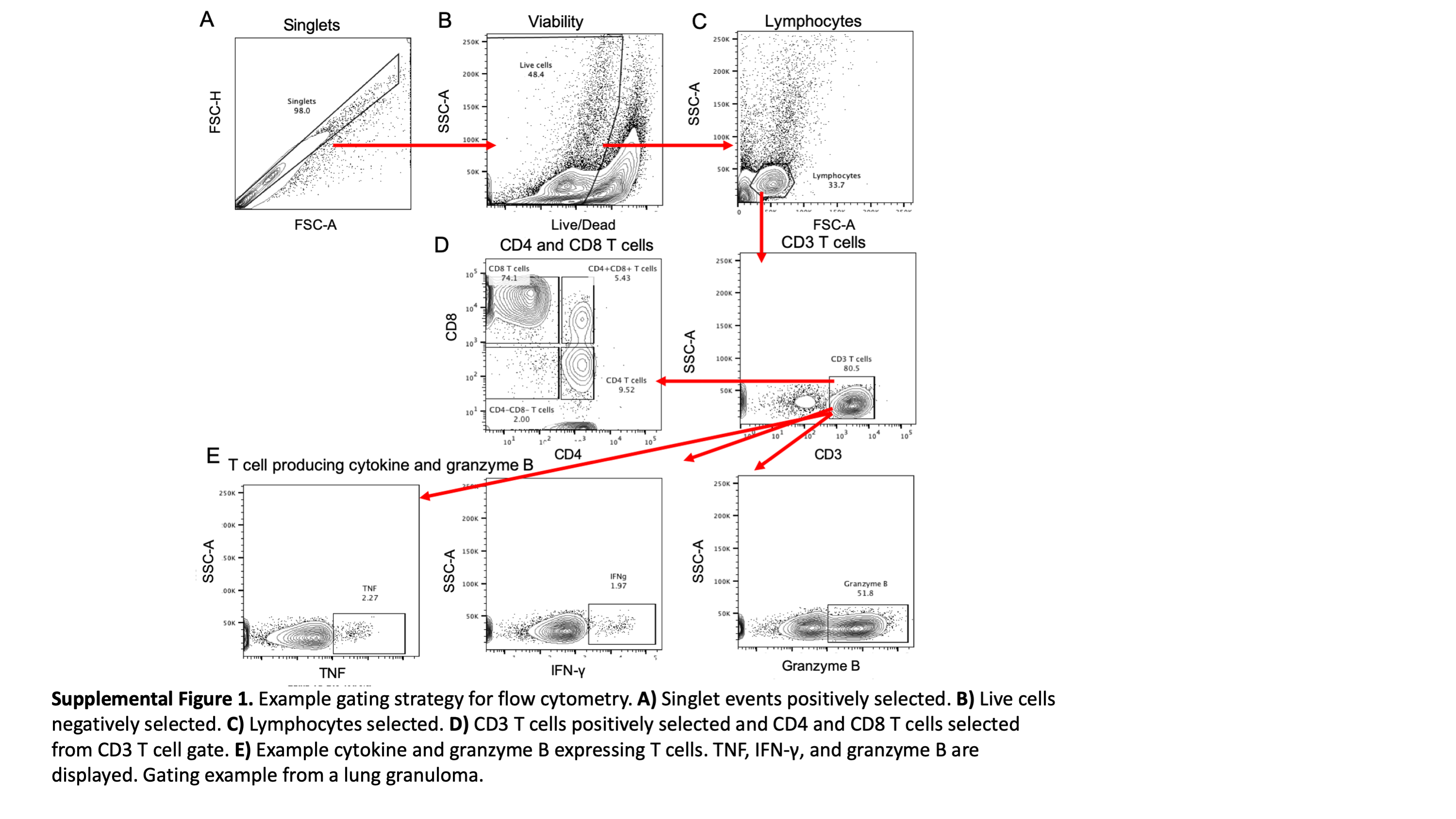

Supplement: S1 Fig — A) Singlet events positively selected. B) Live cells negatively selected. C) Lymphocytes selected. D) CD3 T cells positively selected and CD4 and CD8 T cells selected from CD3 T cell gate. E) Example cytokine and granzyme B expressing T cells. TNF, IFN-γ, and granzyme B are displayed. Gating example from a lung granuloma. (TIFF) [file ppat.1008413.s003.tiff]

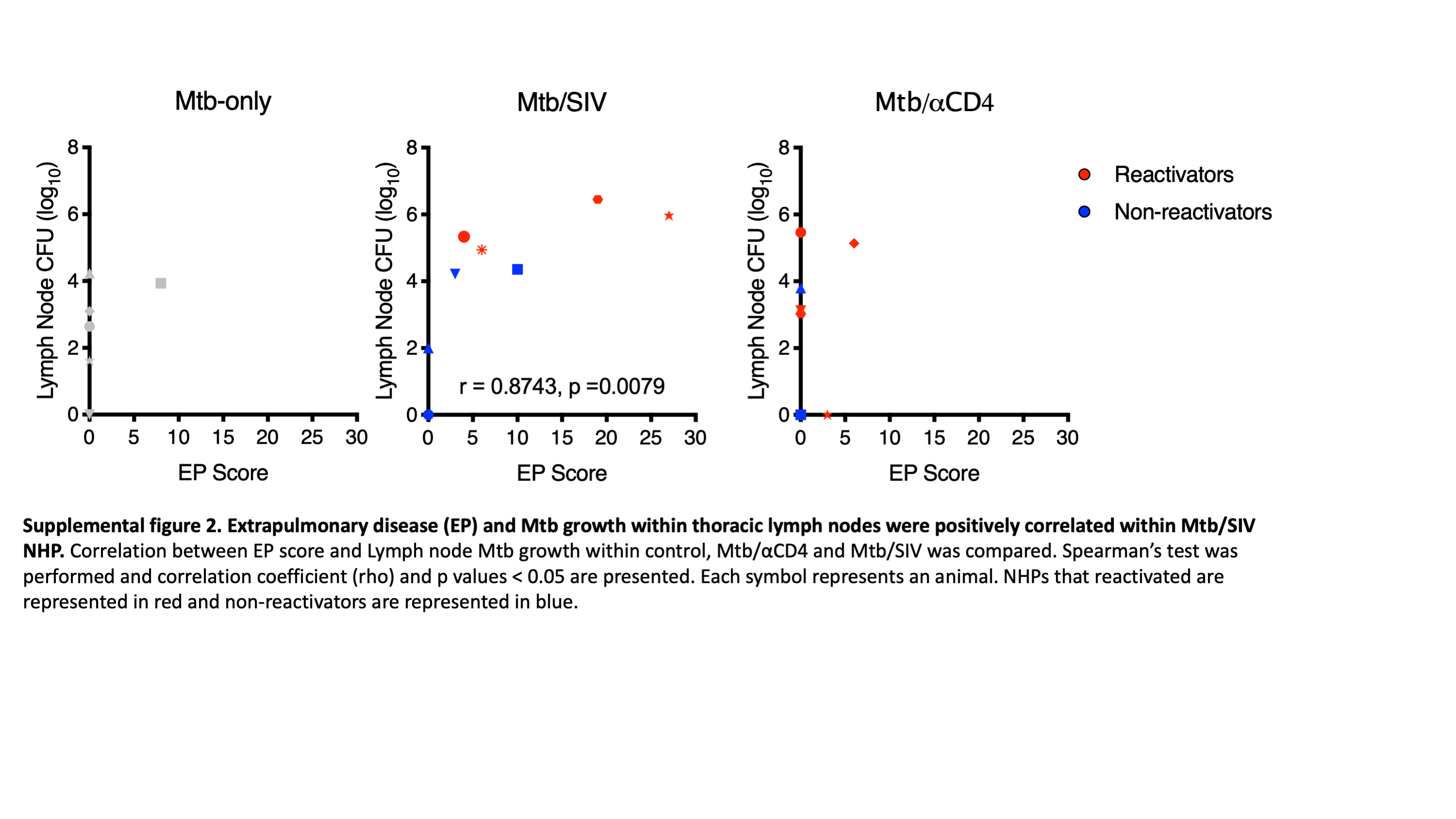

Supplement: S2 Fig — Correlation between EP score and lymph node Mtb growth within Mtb-only, Mtb/αCD4 and Mtb/SIV was compared. Individual monkeys are identified by different shapes. Spearman’s test was performed and correlation coefficient (rho) and p values < 0.05 are presented. Each dot represents an animal. NHPs that reactivated are represented in red and non-reactivators are represented in blue. (TIFF) [file ppat.1008413.s004.tiff]

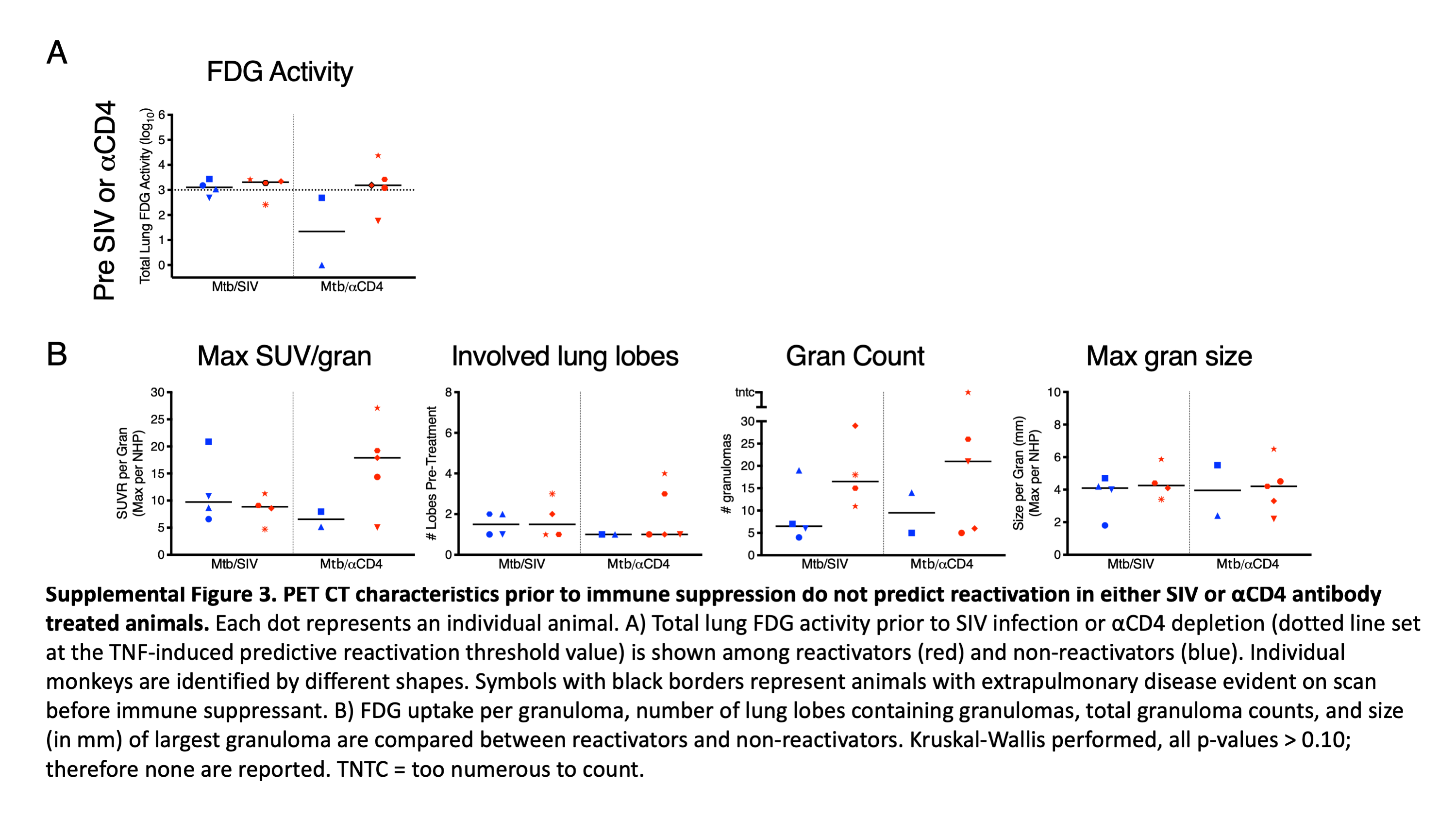

Supplement: S3 Fig — Each dot represents an individual animal. A) Total lung FDG activity prior to SIV infection or αCD4 depletion (dotted line set at the TNF-induced predictive reactivation threshold value) is shown among reactivators (red) and non-reactivators (blue). Individual monkeys are identified by different shapes. Symbols with black borders represent animals with extrapulmonary disease evident on scan before immune suppressant. B) FDG uptake per granuloma, number of lung lobes containing granulomas, total granuloma counts, and size (in mm) of largest granuloma are compared between reactivators and non-reactivators. Kruskal-Wallis performed, all p-values > 0.10; therefore none are reported. TNTC = too numerous to count. (TIFF) [file ppat.1008413.s005.tiff]

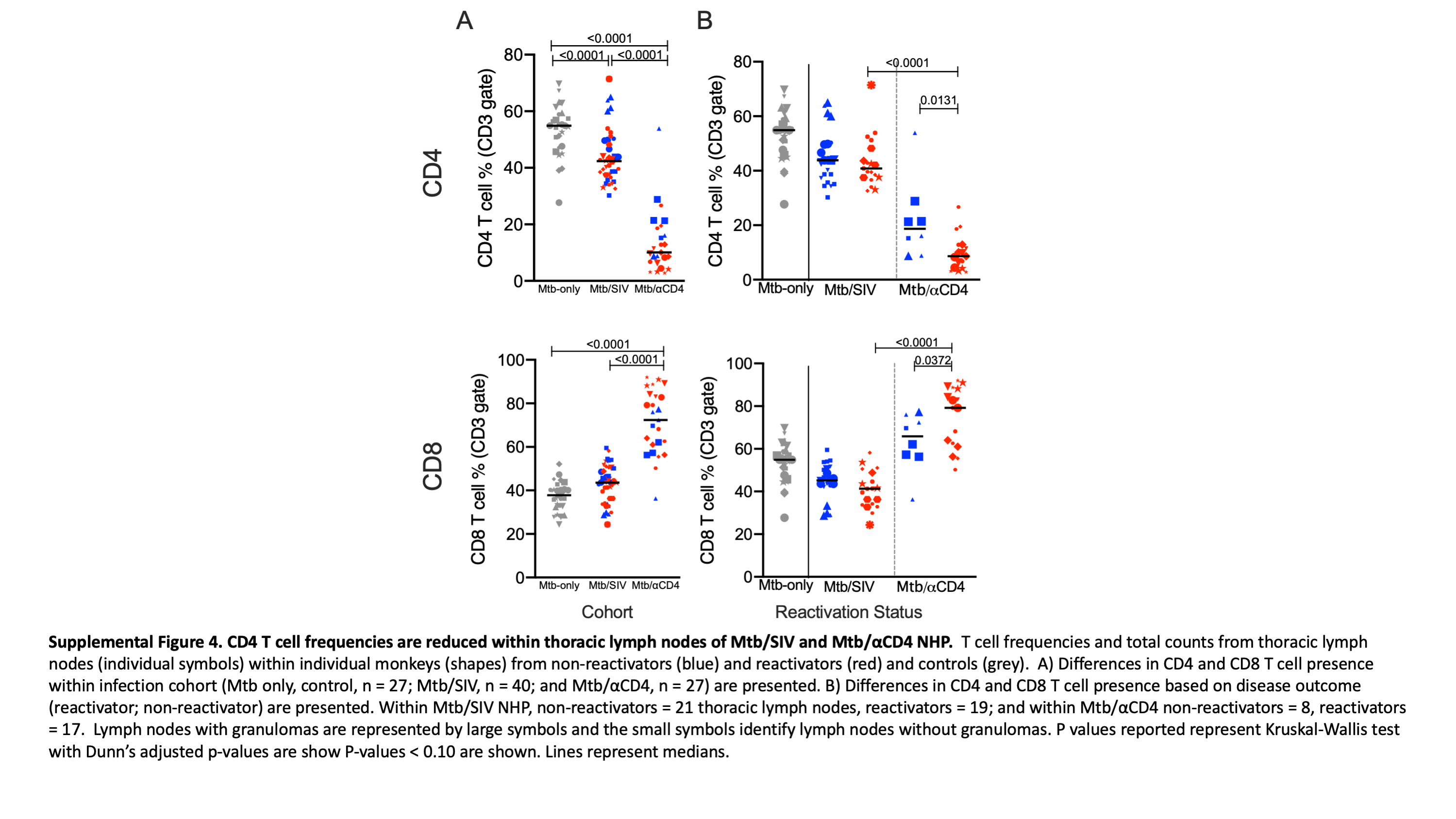

Supplement: S4 Fig — T cell frequencies and total counts from thoracic lymph nodes (individual symbols) within individual monkeys (shapes) from non-reactivators (blue) and reactivators (red) and controls (grey). A) Differences in CD4 and CD8 T cell presence within infection cohort (Mtb only, control, n = 27; Mtb/SIV, n = 40; and Mtb/αCD4, n = 27) are presented. B) Differences in CD4 and CD8 T cell presence based on disease outcome (reactivator; non-reactivator) are presented. Within Mtb/SIV NHP, non-reactivators = 21 thoracic lymph nodes, reactivators = 19; and within Mtb/αCD4 non-reactivators = 8, reactivators = 17. Lymph nodes with granulomas are represented by large symbols and the small symbols identify lymph nodes without granulomas. P values reported represent Kruskal-Wallis test with Dunn’s adjusted p-values are show P-values < 0.10 are shown. Lines represent medians. (TIFF) [file ppat.1008413.s006.tiff]

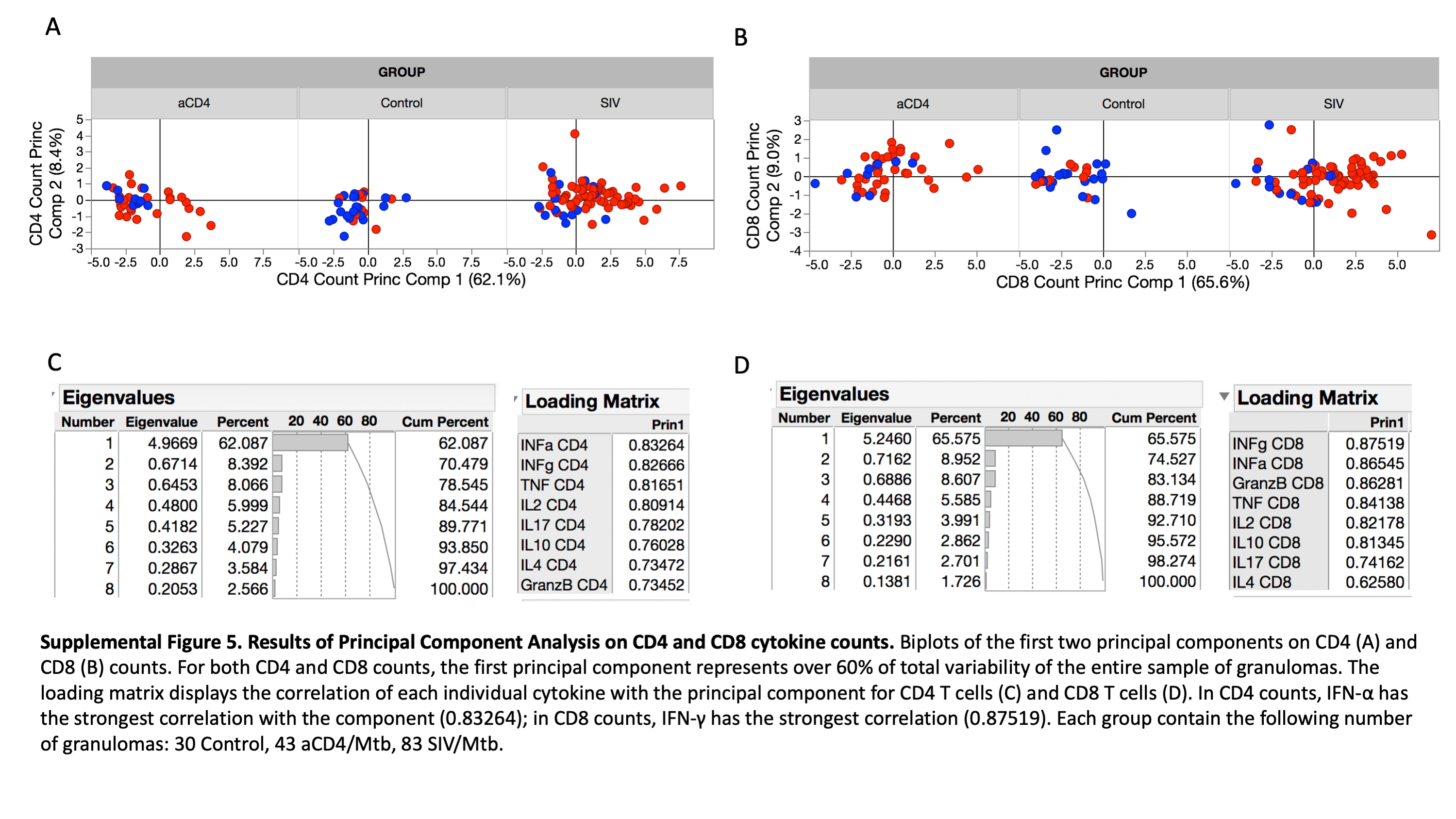

Supplement: S5 Fig — Biplots of the first two principal components on CD4 (A) and CD8 (B) counts. For both CD4 and CD8 counts, the first principal component represents over 60% of total variability of the entire sample of granulomas. The loading matrix displays the correlation of each individual cytokine with the principal component for CD4 T cells (C) and CD8 T cells (D). In CD4 counts, IFN-α has the strongest correlation with the component (0.83264); in CD8 counts, IFN-γ has the strongest correlation (0.87519). Each group contain the following number of granulomas: 30 Control, 43 Mtb/αCD4., 83 Mtb/SIV. (TIFF) [file ppat.1008413.s007.tiff]

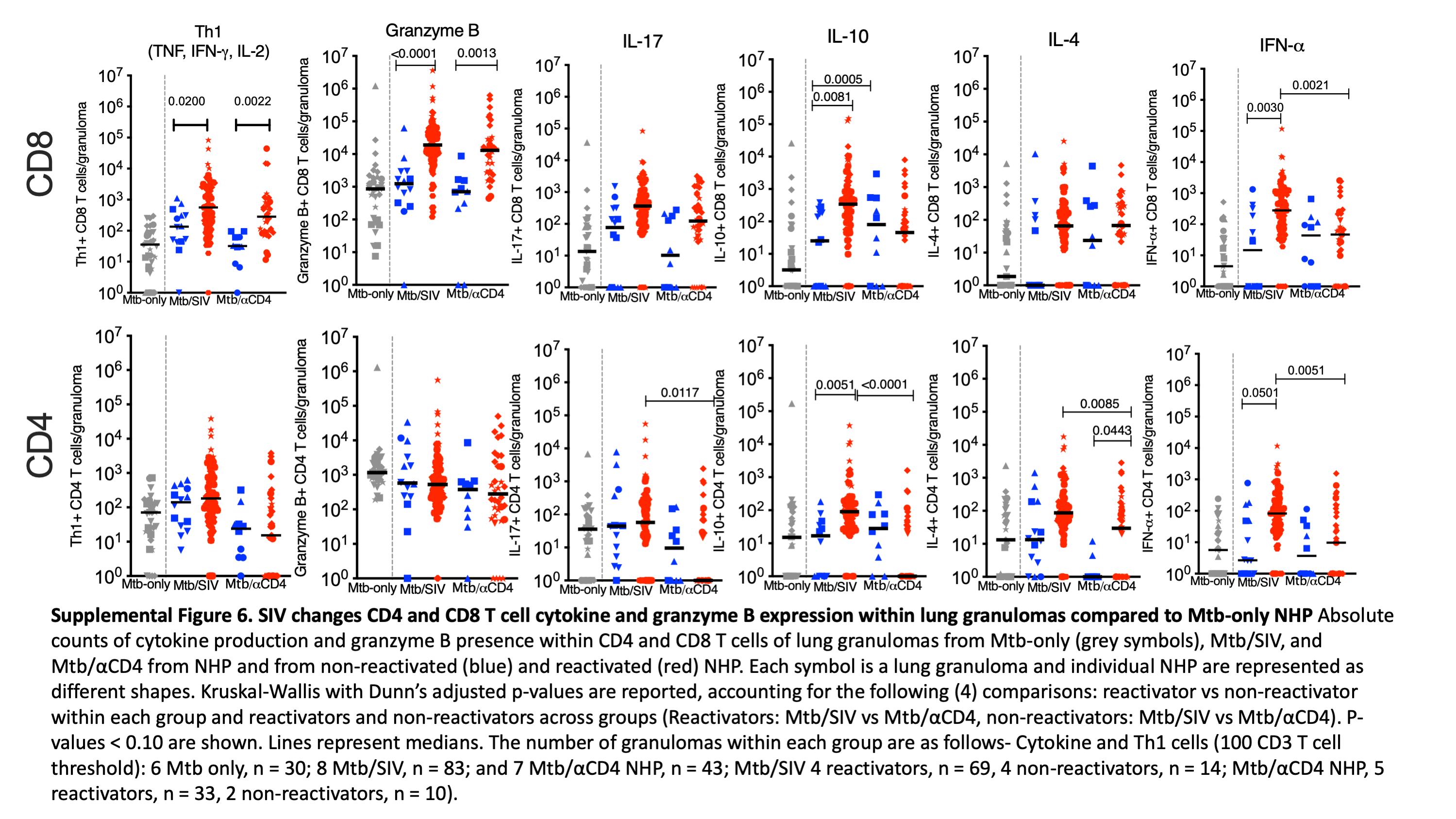

Supplement: S6 Fig — Absolute counts of cytokine production and granzyme B presence within CD4 and CD8 T cells of lung granulomas from Mtb-only (grey symbols), Mtb/SIV, and Mtb/αCD4 NHP and from non-reactivated (blue) and reactivated (red) NHP. Each symbol is a lung granuloma and individual NHP are represented as different shapes. Kruskal-Wallis with Dunn’s adjusted p-values are reported, accounting for the following (4) comparisons: reactivator vs non-reactivator within each group and reactivators and non-reactivators across groups (Reactivators: Mtb/SIV vs Mtb/αCD4, non-reactivators: Mtb/SIV vs Mtb/αCD4). P-values < 0.10 are shown. Lines represent medians. The number of granulomas within each group are as follows- Cytokine and Th1 cells (100 CD3 T cell threshold): 6 Mtb only, n = 30; 8 Mtb/SIV, n = 83; and 7 Mtb/αCD4 NHP, n = 43; Mtb/SIV 4 reactivators, n = 69, 4 non-reactivators, n = 14; Mtb/αCD4 NHP, 5 reactivators, n = 33, 2 non-reactivators, n = 10). (TIFF) [file ppat.1008413.s008.tiff]

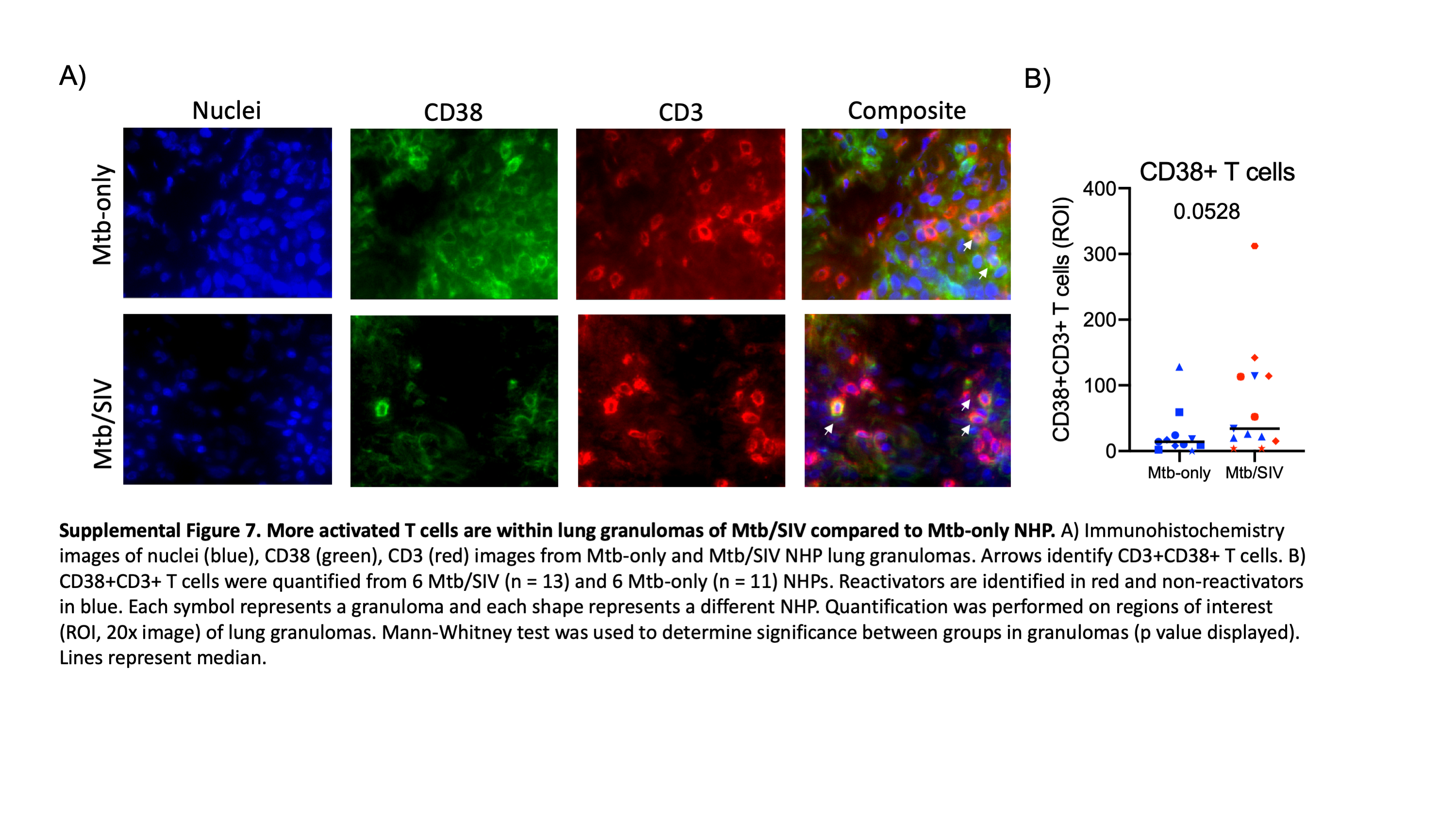

Supplement: S7 Fig — A) Immunohistochemistry images of nuclei (blue), CD38 (green), CD3 (red) from Mtb-only and Mtb/SIV NHP lung granulomas. Arrows identify CD3+CD38+ T cells. B) CD38+CD3+ T cells were quantified from 6 Mtb/SIV (n = 13) and 6 Mtb-only (n = 11) NHPs. Reactivators are identified in red and non-reactivators in blue. Each symbol represents a granuloma and each shape represents a different NHP. Quantification was performed on regions of interest (ROI, 20x image) of lung granulomas. Mann-Whitney test was used to determine significance between groups in granulomas (p value displayed). Lines represent median. (TIFF) [file ppat.1008413.s009.tiff]

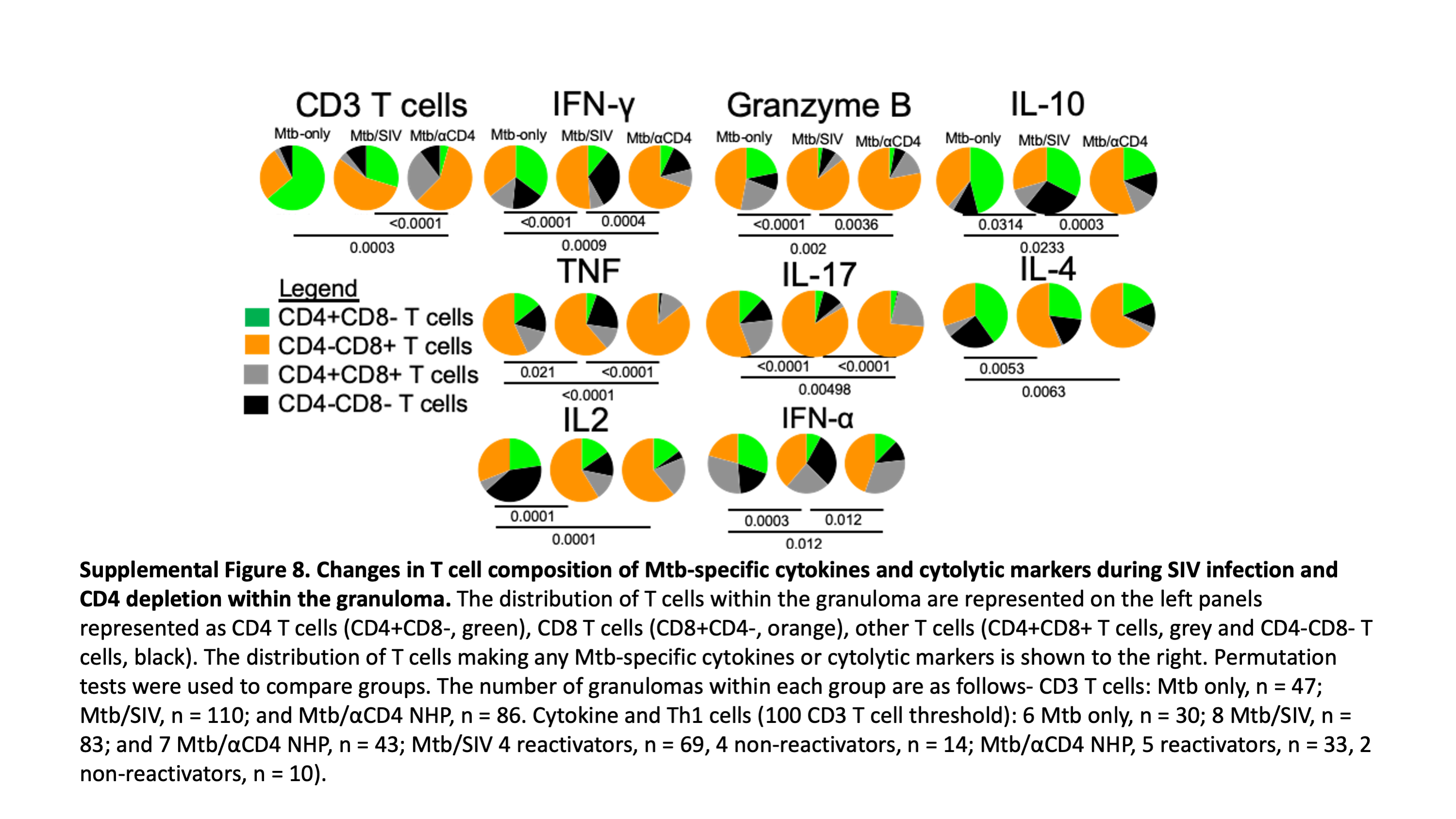

Supplement: S8 Fig — The distribution of T cells within the granuloma are represented on the left panels represented as CD4 T cells (CD4+CD8-, green), CD8 T cells (CD8+CD4-, orange), other T cells (CD4+CD8+ T cells, grey and CD4-CD8- T cells, black). The distribution of T cells making any Mtb-specific cytokines or cytolytic markers is shown to the right. Permutation tests were used to compare groups. The number of granulomas within each group are as follows- CD3 T cells: Mtb only, n = 47; Mtb/SIV, n = 110; and Mtb/αCD4 NHP, n = 86. Cytokine and Th1 cells (100 CD3 T cell threshold): 6 Mtb only, n = 30; 8 Mtb/SIV, n = 83; and 7 Mtb/αCD4 NHP, n = 43; Mtb/SIV 4 reactivators, n = 69, 4 non-reactivators, n = 14; Mtb/αCD4 NHP, 5 reactivators, n = 33, 2 non-reactivators, n = 10). (TIFF) [file ppat.1008413.s010.tiff]

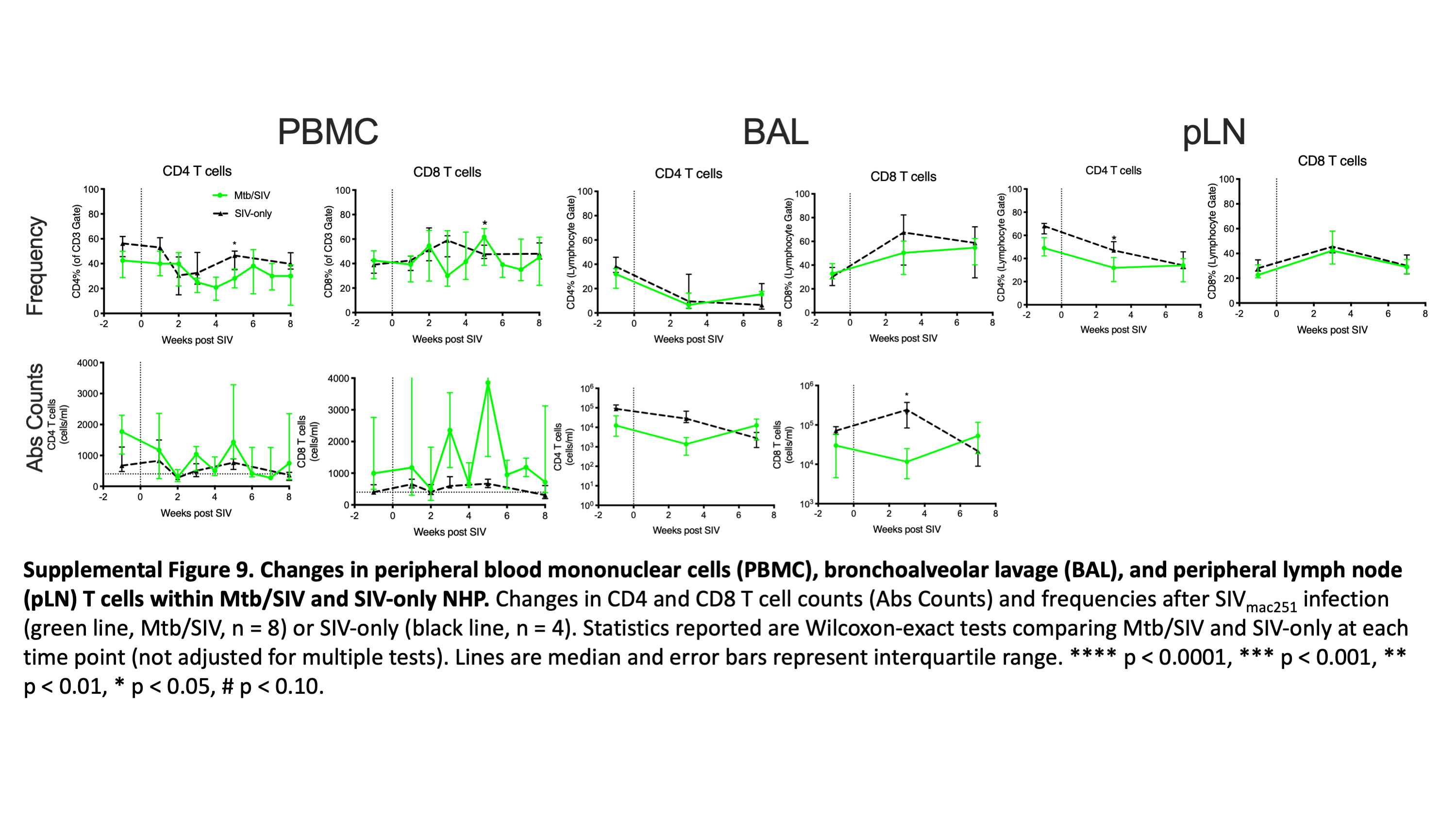

Supplement: S9 Fig — Changes in CD4 and CD8 T cell counts (Abs Counts) and frequencies after SIVmac251 infection (green line, Mtb/SIV, n = 8) or SIV-only (black line, n = 4). Statistics reported are Wilcoxon-exact tests comparing Mtb/SIV and SIV-only at each time point (not adjusted for multiple tests). Lines are median and error bars represent interquartile range. **** p < 0.0001, *** p < 0.001, ** p < 0.01, * p < 0.05, # p < 0.10. (TIFF) [file ppat.1008413.s011.tiff]

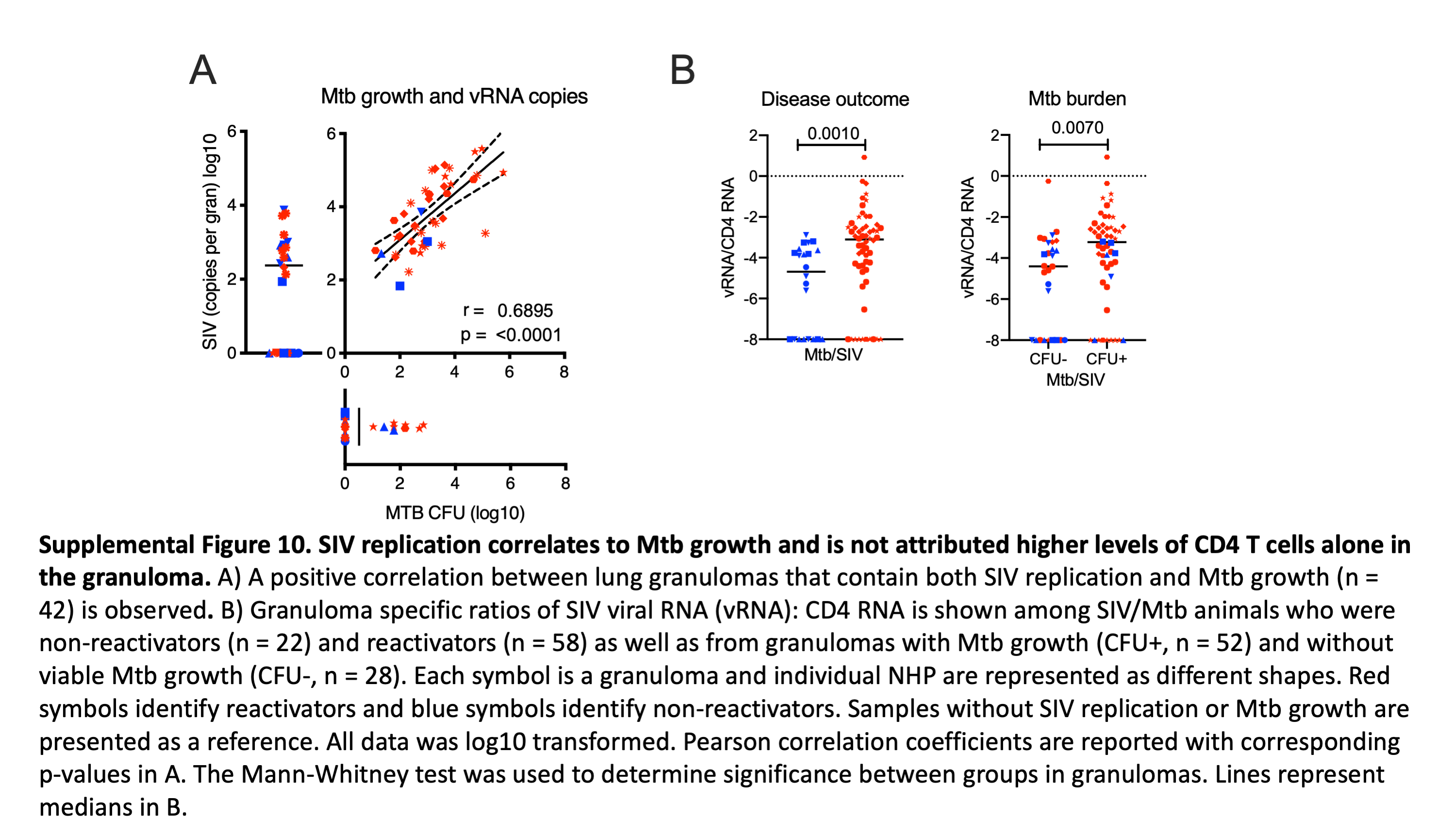

Supplement: S10 Fig — A) A positive correlation between lung granulomas that contain both SIV replication and Mtb growth (n = 42) is observed. B) Granuloma specific ratios of SIV viral RNA (vRNA): CD4 RNA is shown among SIV/Mtb animals who were non-reactivators (n = 22) and reactivators (n = 58) as well as from granulomas with Mtb growth (CFU+, n = 52) and without viable Mtb growth (CFU-, n = 28). Each symbol is a granuloma and individual NHP are represented as different shapes. Red symbols identify reactivators and blue symbols identify non-reactivators. Samples without SIV replication or Mtb growth are presented as a reference. All data was log10 transformed. Pearson correlation coefficients are reported with corresponding p-values in A. The Mann-Whitney test was used to determine significance between groups in granulomas. Lines represent medians in B. (TIFF) [file ppat.1008413.s012.tiff]
